# Mini review: Targeting below-ground plant performance to improve nitrogen use efficiency (NUE) in barley

**DOI:** 10.3389/fgene.2022.1060304

**Published:** 2023-03-02

**Authors:** Claire Huang, Clayton R. Butterly, David Moody, Mohammad Pourkheirandish

**Affiliations:** ^1^ Faculty of Veterinary and Agricultural Sciences, The University of Melbourne, Melbourne, VIC, Australia; ^2^ InterGrain Pty Ltd., Bibra Lake, WA, Australia

**Keywords:** nitrogen use efficiency, genome-wide association study, root architecture, nitrogen transport, root phenotyping

## Abstract

Nitrogen (N) fertilizer is one of the major inputs for grain crops including barley and its usage is increasing globally. However, N use efficiency (NUE) is low in cereal crops, leading to higher production costs, unfulfilled grain yield potential and environmental hazards. N uptake is initiated from plant root tips but a very limited number of studies have been conducted on roots relevant to NUE specifically. In this review, we used barley, the fourth most important cereal crop, as the primary study plant to investigate this topic. We first highlighted the recent progress and study gaps in genetic analysis results, primarily, the genome-wide association study (GWAS) regarding both biological and statistical considerations. In addition, different factors contributing to NUE are discussed in terms of root morphological and anatomical traits, as well as physiological mechanisms such as N transporter activities and hormonal regulation.

## 1 Introduction

N fertilizer is the primary resource to supply soil nutrients to conserve cereal crop production (Raun & Johnson, 1999; Sadras et al., 2016). N fertilization input is increasing globally while the global average for N recovery rate in cereal crops is only 33% (Raun & Johnson, 1999). Un-utilized N fertilizers are likely cause environmental hazards, such as eutrophication, soil acidification, and air pollution (Raun & Johnson, 1999; Tang et al., 2013; Skiba & Rees, 2014). NUE itself has barely been considered in most modern breeding programs and a continuous selection of high-yielding genotypes only under sufficient N environments has led to the reduced variation of NUE alleles in modern varieties (Yang et al., 2014; Garnett et al., 2015).

Barley (*Hordeum vulgare*) is an important cereal and ranks the fourth most grown crop worldwide (Arendt & Zannini, 2013). Barley is one of the most ancient cereal crops used by hunters and gatherers, and the history of its cultivation can be traced back to more than 20,000 years ago (Willcox, 2013; Pourkheirandish et al., 2015). In addition to its importance as a crop, the diploid nature of barley, the availability of multiple reference quality genomes, reference transcriptome, pangenome sequence, and its close genetic relationship with wheat, make it an ideal model for cereal crops (von Bothmer et al., 2003). Once a molecular mechanism relevant to the expression of a specific trait associated with NUE is discovered, it can be expected to offer opportunities for crop improvement both in barley and its close relatives.

Genome-wide association study (GWAS) is often referred as a “hypothesis-generating” analysis and could serve as a first step to gaining novel understanding of mechanisms of NUE by studying genetic variations of the entire genome instead of only targeting specified genes of interest (Ali et al., 2021; Özdemir et al., 2021). Although numerous above-ground crop traits have been determined to be closely correlated with NUE as well as identifying associated genes conferring high NUE in pre-breeding research (Yang et al., 2014; Han et al., 2016; Karunarathne et al., 2020a), a great study gap is present in relevant below-ground activities due to difficulties in phenotyping root traits under field conditions and dynamics in soil N status (Garnett et al., 2009; Plett et al., 2020). This mini-review will report the recent progress in identifying NUE-related genetic variants and phenotypic traits in barley as well as discuss the limitations and knowledge gap.

## 2 Genome-wide association studies in nitrogen use efficiency

GWAS is a powerful tool to examine the contributions of genetic variants/single nucleotide polymorphisms (SNPs) to the traits of interests (Bush, 2019; Alqudah et al., 2020; Alomari et al., 2021). NUE is a polygenic trait which has not had its molecular mechanisms fully revealed, resulting in difficulties to target genes of interest in pre-breeding research. The rapid progress in high-throughput genomic technologies such as next-generation sequencing (NGS) has allowed more accurate reads for whole genome sequencing data, subsequently facilitating the precision of results generated from GWAS (Wu et al., 2017; Yin et al., 2019). Non-etheless, there is only one published research on examining NUE in barley to date (Karunarathne et al., 2020a). Most of the genes associated with NUE in barley were reported by quantitative trait loci (QTL) analysis without being well correlated with phenotypic traits. Although, only root length and dry weight have been examined for NUE in barley by GWAS (Supplementary Table S1), it has provided reliable and important genetic information to associate root traits and NUE. For example, HORVU3Hr1G095880 is the candidate gene associated with root dry weight and functionally annotated as NAC domain protein. The role of NAC domain protein on NUE has been well demonstrated by overexpression it in wheat and the improvement in several agronomic traits have been shown in both low and high N levels (He et al., 2015). Further investigation into identifying QTLs associated with various root traits would be valuable to discover novel mechanisms of NUE.

### 2.1 Statistical considerations for genome-wide association study and meta-analysis

Several limitations of GWAS impede the implication of GWAS results for improving NUE. One of the major issues is that the identified variants are often unstable across experiments (Karunarathne et al., 2020b). There is a demand to increase statistical power of GWAS to generate replicable variants and the most direct approach is to incorporate a bigger sample size into the study. Another issue is that the detected variants can be false positives due to the complex population structures and cryptic relatedness (Wright et al., 2012; Yang et al., 2021). To date, fitting the population structure and/or kinship matrix as covariate to adjust the mixed linear model and assessing linkage disequilibrium (LD) block to project candidate genes are the most utilized methods to pinpoint the relevant variants (Kovi et al., 2015; Xu et al., 2017; Karunarathne et al., 2020a). Nonetheless, false negatives are caused at the same time when attempting to control those false positives.

Computing innovation has allowed a more powerful GWAS by increasing the statistical power as well as optimizing the detection of accurate variants. An early GWAS model, described as a univariate model, can only analyze a single phenotype for its association with variants (Fernandes et al., 2021). This model poses limitation of taking account of relatedness among phenotypic traits while most loci are pleiotropic. For instance, NUE is under the regulation of multiple genes, and each can affect several phenotypic traits associated with NUE so that a univariate GWAS could mask some information of the phenotypic and genetic relatedness. Subsequently, the multivariate model of GWAS was developed to improve the statistical power for detecting variants controlling multiple phenotypic traits and reducing false discoveries (Korte et al., 2012). Both models would also compromise partial true positives due to the confounding effect among the variants, population structure and kinship since they principally still utilize the same mixed linear model to adjust population structure and LD (Liu et al., 2016).

Although a few advancements have been achieved, the optimization of both statistical power and computational efficiency when controlling false discoveries was the primary burden. For instance, fixed and random model Circulating Probability Unification (FarmCPU) was developed to resolve the confounding issue owing to population structure and kinship by dissecting the model into fixed effect model and random effect model to separate population structure and kinship, then running the two models iteratively (Liu et al., 2016). Thereafter FarmCPU boosted the statistical power while the computing speed was the primary issue to be tackled. Bayesian-information and Linkage-disequilibrium Iteratively Nested Keyway (BLINK) is the new method built based on FarmCPU. BLINK utilizes Bayesian Information Criteria to substitute the computationally demanding random effect model into the fixed effect model and has been tested with its precision and efficiency with both simulated and real datasets (Huang et al., 2018). BLINK model has been employed in several research investigating plant performance such as rice, wheat and barley (Kumar et al., 2021; Zhong et al., 2021; Clare et al., 2022). Moreover, BLINK GWAS model has also be used to examine maize root performance in different N levels (Fu et al., 2022). Accordingly, BLINK would be the most updated GWAS model for ascertaining and providing context to the genetic-phenotypic relationship for plant underground performance in various N environments.

Meta-analysis of GWAS (Meta-GWAS) is another model worthen attention. It is to analyze the quantitative combination of summary statistics from independent experiments (Joukhadar and Daetwyler, 2022). Subsequently, it can increase sample size by analyzing multiple GWAS results, which boost statistical power (Lin and Zeng, 2010). Moreover, the algorithm of Meta-GWAS has been developed to effectively detect variants associated with multiple traits and environments (Bolormaa et al., 2014; Singh et al., 2021). NUE is affected by multiple traits and N availability is varied greatly from different environments due to its mobile nature. Therefore, meta-analysis can be useful for studying NUE and detecting more associated variants by analyzing different combination of associated traits and examining several N levels at the same time. Another advantage of meta-analysis is its capability to incorporate data from various genetic backgrounds (Tam et al., 2019). It could potentially bring extra benefits to increase the magnitude of association of the determined variants when significant association between variants and population structure presents (Tam et al., 2019). This provides insight to interpret the results with sound consideration of population structure and LD.

### 2.2 Biological considerations for genome-wide association study and relevant phenotyping platforms

The accuracy of GWAS counts upon both high density markers and precise phenotyping. With the advancement in genome sequencing and availability of reference genome, the high-density genotyping is no longer a limit. Our understanding on the plant genome has been more advanced than the below-ground phenome in regards of barley NUE (Yang et al., 2014; Karunarathne et al., 2020a). Therefore, the information on the relationship between root traits and NUE could be ambiguous and lead to misinterpretation of data. This becomes an impediment for pre-breeding research of NUE even if molecular tools are well-developed.

Growing conditions have enormous impacts on root growth and development while there are different growth systems in pre-breeding research (Zhu et al., 2005; Liao et al., 2006; Karunarathne et al., 2020a). The results concluded from one growth system are not necessarily replicable in another one. For instance, a comparative study on maize has been conducted in three systems, which are paper roll, hydroponics, and vermiculite culture. The results demonstrated that only 13% of QTLs were repeatably identifiable across systems due to strong genetic-environmental effects (Liu et al., 2017). Moreover, this study also determined a relatively stronger correlation between root traits and N uptake efficiency in hydroponics and vermiculite systems compared to the paper roll system, suggesting they are the more suitable growth systems to examine NUE in maize (Liu et al., 2017).

The development of high-throughput phenotyping technologies is paramount for root phenotyping since the sample size for detecting QTLs is generally large and processing those samples in conventional ways can be laborious and time-consuming. The most common method is to collect root samples by washing them from soils and then scan clean samples using scanning software such as WinRhizo and RhizoVision (Kamoshita et al., 2019; Seethepalli et al., 2021). One of the difficulties during this process is efficiently getting the root out from fields. A method described by Teramoto et al. (2019) is to place a steel cylinder surrounding each plant then the whole plant can be pushed back vertically by a backhoe. It is demonstrated to greatly reduce labor work. Non-etheless, root washing processes are still challenging regarding labor intensity and sample integrity. The development of X-ray computed tomography (CT) permits the visualization of root in the soils so that root traits data could be collected non-destructively, which not only reduces the labor requirement but also guarantee the quality of data (Teramoto et al., 2020). This technology has been utilized in various crops, such as rice, wheat, and maize, for visualizing root systems in soils (Mairhofer et al., 2012; Teramoto et al., 2020). However, no barley NUE research so far has utilized this platform to examine root traits in soils.

## 3 Morphological and anatomical traits of roots contributing to nitrogen use efficiency

### 3.1 Root biomass

It is a widely accepted concept in cereals that larger root biomass can enhance N absorption (Anbessa & Juskiw, 2012). Root weight density refers to total root weight per unit of soil volume, which has been found to be significantly correlated with NUE of wheat (Liu et al., 2018). However, it does not always translate into better above-ground performance. First, the trade-off between above- and below-ground carbon (C) storage could limit the yield capacity when large amounts of energy are invested into root biomass. Several studies have supported this trade-off effect in maize and barley when N is insufficient (Gallais and Coque, 2005; Lynch, 2013; Tolley and Mohammadi, 2020). In contrast, research on wheat has indicated that larger root biomass is associated with higher yield and less leached N in high N conditions (Ehdaie & Waines, 2008; Tolley & Mohammadi, 2020). It implies that the yields are not being limited by the higher metabolic costs of roots since nutrient resources are sufficient to supply both above- and below-ground plant growth. To connect with above-ground performance, root:shoot (R:S) ratio is often used to explain the energy allocation. Research has suggested that R:S are altered by different N levels as the strategy to optimize the relative growth rate (Ågren & Franklin, 2003), but it has not been shown if R:S has a significantly linear association with NUE.

### 3.2 Root system architecture

Root system architecture (RSA) is a collection of root features to describe the temporospatial distribution of roots in the soil. Intensive investigations have been done for the root length and it is positively related to N capture (Karunarathne et al., 2020a). With the same root length, it is desirable to obtain other RSA traits for optimizing the interception between root and soil while minimizing metabolic costs. One of the strategies could be targeting the specific root zones for nutrient uptake (Kitomi et al., 2018). The combination of the narrow root angle and deep roots, or the wide root angle and shallow roots have been suggested to enable reducing metabolic costs while maximizing soil nutrient exploitation in deeper and shallow soil profiles, respectively (Lynch, 2013). However, no significant correlation between root angle and barley grain yield has been determined so far, demanding more studies to map the correlation between root angle and NUE (Robinson et al., 2018). For a further step, increased lateral root number and root hairs, subsequently root surface area, have been found when plants are grown in N-deficient conditions (Marschner 1995; Gilroy & Jones, 2000). The debate has been around if such responses would contribute to N uptake when sufficient N is supplied to plants, specifically in the soil with a low leaching potential where N is abundant and the increase in root surface area is not required (Garnett et al., 2009). Meanwhile, it has also been reported that wheat with a higher level of root branching and lateral root number showed greater N uptake in sandy soils with an ample N level (Liao et al., 2006). Accordingly, the N availability and the leaching potential of soils are vital to be considered to conclude if increased root surface area is of importance for increasing N uptake.

### 3.3 Root cortical senescence

Root cortical senescence (RCS) describes the death of root cortical cells, which can be promoted by severe N scarcity (Gillespie & Deacon, 1988). On the one hand, this could result in unhealthy plant tissues and restricted plant growth (Schneider et al., 2017a). On the other hand, RCS might demonstrate potential compensation for a plant’s survival in harsh environments. Initially, a reduction in nutrient partitioning, and efficiencies of radial water and nutrient transport in barley root have been determined to be correlated with RCS under N-deficient conditions (Schneider et al., 2017a). Simultaneously, barley root respiration also decreases, which can infer as a positive effect of RCS, saving the energy cost of roots (Schneider et al., 2017b). Also, RCS occurs in older tissues, located in the upper part of soil profile where nutrients might be depleted (Schneider et al., 2017a; Schneider et al., 2017b). Hence, it can be beneficial for young tissue growth when coming across nutrient-deficient situations since energy can be reinvested into effective plant parts by getting rid of older tissues (Schneider et al., 2017b).

### 3.4 Importance of nodal roots

Cereals have a fibrous root system that can be mainly dissected into two parts, seminal and nodal roots. Nevertheless, much less attention has been paid to nodal roots for their role in NUE until recently. A greater root diameter, meta-xylem area and more meta-xylem vessels have been examined in nodal roots compared to seminal roots in mature barley plants, which also contributes to a higher level of nitrate (NO_3_^−^) uptake (Liu B. et al., 2020). Hence, nodal roots may serve as a selection target in pre-breeding research for better barley NUE. Nevertheless, no similar analysis has been done on barley from the seedling stage. It might be since nodal roots have been considered more important for water uptake at lateral plant stages (Hochholdinger et al., 2018), and this concept has been applied to N uptake as well. Meanwhile, the work done by Schneider et al. (2020) showed barley nodal roots demonstrated equivalent importance of water uptake as seminal roots since the seedling stage. Given that N flux into plant roots with water via mass flow, this study enlightens the possibility that nodal roots might also be important in N uptake from an early stage.

## 4 Physiological aspects relevant to nitrogen use efficiency

### 4.1 Nitrogen transporter activities and metabolism of different N forms

**FIGURE 1:**
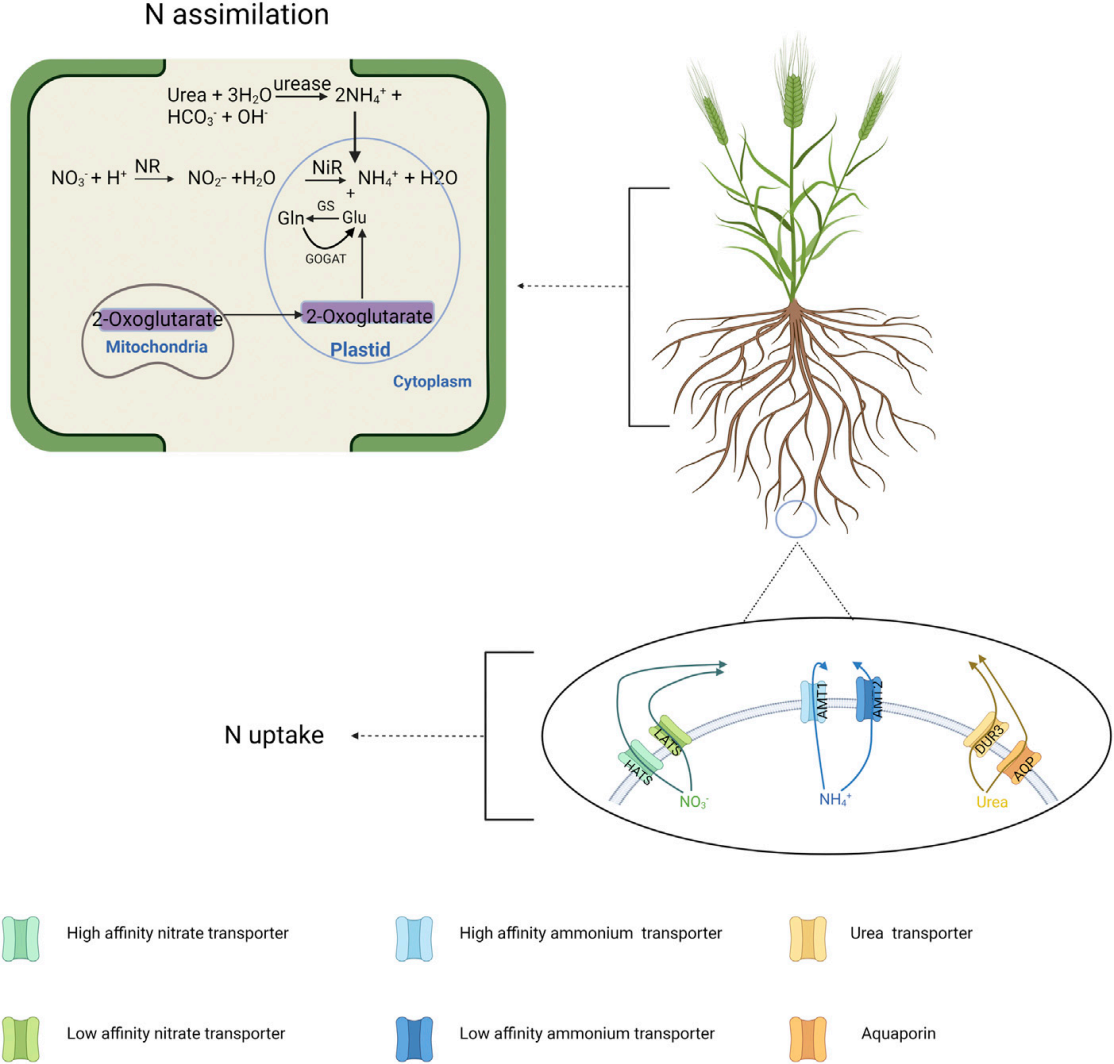
N transport and assimilation in the barley plant at a cellular level. In the transport process, different forms of N including nitrate, ammonium and urea are transported *via* different classes of proteins in different concentrations. In the assimilation process, the conversion of nitrate and urea into ammonium is happened first, followed by GS-GOGAT cycle to convert ammonium into glutamine (Gln) and glutamate (Glu). Created with BioRender.com.

N molecules pass through the plasma membrane of root cells, initiating the N uptake process, mediated by several N transporters (Tsay & Hsu, 2010; Hove et al., 2015; Han et al., 2016; Figure 1). Nitrate (NO_3_^−^) is widely considered as the most common form absorbed by crops since nitrification is continuously taking place in the soil under the effect of soil bacteria, converting ammonium (NH_4_^+^) into NO_3_^−^. Therefore, most research has focused on NO_3_^−^. After NO_3_^−^ is taken up by roots, it is reduced into nitrite (NO_2_^−^) by NO_3_^−^ reductase (NR), then further reduced into NH_4_^+^ by NO_2_^−^ reductase (NiR). Subsequently, N in the NH_4_^+^ form will undergo several enzymatic reactions and be converted into organic forms, predominantly amino acids (Tsay & Hsu, 2010). To illustrate, glutamate and glutamine are synthesized in the plastid, which are the two primary sources of the amine group for other amino acids (Tsay & Hsu, 2010; Han et al., 2016). This process is primarily regulated by two enzymes, which are glutamine synthetase (GS) and glutamate synthetase (GOGAT) (Tsay & Hsu, 2010; Han et al., 2016). Moreover, it has been determined that NO_3_^−^ has an important role in regulating lateral root growth (Zhang & Forde, 2000; Liu B. et al., 2020).

NH_4_^+^ has often been overlooked due to its lower availability in soils and adverse effects on several domesticated plants including barley (Britto & Kronzucker, 2002; Liu et al., 2013; Chen et al., 2020; Naz et al., 2021). Interestingly, wheat cultivars, Yitpi and Wyalkatchem, have been determined to prefer NH_4_^+^ over NO_3_^−^ (O’Sullivan et al., 2016). Moreover, NH_4_^+^ can become more significant in certain environments including low N conditions, and soil environments unfavored by nitrification. Initially, crops will preferentially absorb NH_4_^+^ in nutrient-depleted soils, since unlike NO_3_^−^ it does not need to undergo energy-dependent transformation once inside the root (Boudsocq et al., 2012). Further, soil acidification and insufficient soil organic matter could inhibit bacterial processes, such as nitrification. Subsequently, NH_4_^+^ would become more abundant since less NH_4_^+^ would be converted into NO_3_^−^. Accordingly, selecting barley genotypes for better use of NH_4_^+^ could improve its NUE performance in field conditions featured by N deficiency, acidity, and low organic matter contents. Nevertheless, differing from NO_3_^−^ uptake, a high accumulation of NH_4_^+^ is toxic to barley. Therefore, a more effective use of NH_4_^+^ additionally requires plants to have improved detoxifying mechanisms. It has been shown in barley that NH_4_^+^ toxicity is caused by a great level of NH_4_^+^ efflux across the cell membrane which consumes a huge amount of metabolic energy (Britto et al., 2001). Hence, more efficient utilization of NH_4_^+^ into amino acids *via* GS-GOGAT cycle mentioned above could potentially work as a detoxifying process by reducing NH_4_^+^ accumulation and efflux while also improving overall N utilization into nutrients.

Urea is an organic form of N and is intensively used as N fertilizer in agricultural fields. Urea is hydrolyzed into NH_3_ and ammonium carbamate (H_2_NCOONH_4_), and this process is catalyzed by urease (Figure 1). Although only a limited number of studies have yielded the role of urea itself on plant NUE since the major opinion is that urea uptake by plants is not significant in the past, today’s studies have established several classes of proteins capable to absorb and transport urea (Witte, 2011). For instance, urea transporter (DUR3) and aquaporins (AQP) have been found to act in active and passive urea transport of several plants such as Arabidopsis, maize and barley (Liu et al., 2003; Zanin et al., 2014; Hove et al., 2015). It might offer crops the opportunity to absorb more N with more efficient urea transport while also reducing urea losses as NH_3_ since more urea uptake occurs before being converted into gases.

### 4.2 Hormonal regulation to communicate below-ground with above-ground plant responses: Cytokinin as an example

**FIGURE 2:**
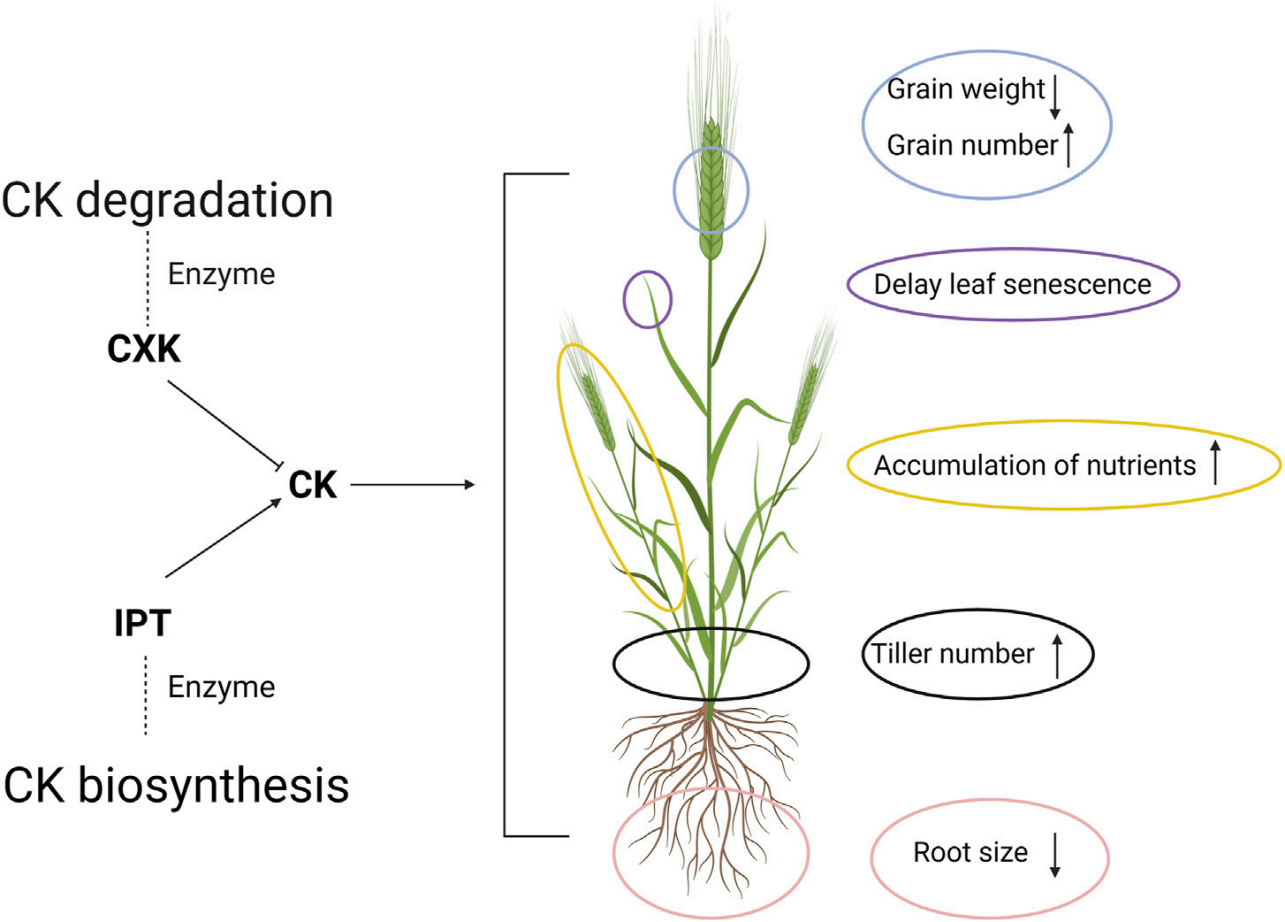
The regulatory role of cytokinin (CK) in the barley plant. The degradation and biosynthesis of CK is majorly controlled by two enzymes, CK oxidase and phosphate-isopentenyl transferase (IPT). The increase in CK level will lead to a series changes of above-ground plant performance associated with N metabolism. The upwards arrows and downwards arrows indicate the increase and decrease in the corresponding traits, respectively. Created with BioRender.com.

Cytokinin (CK) is a plant hormone responsible for plant cell division, which has been suggested as a key substance communicating N availability with plants and coordinating the metabolism from roots to shoots (Lu et al., 1990). The responses of barley relevant to NUE in accordance with CK varied between different plant organs (Figure 2). Two main enzymes, namely phosphate-isopentenyl transferase (IPT) and cytokinin oxidase (CXK) are responsible for CK synthesis and degradation, respectively (del Mar Rubio-Wilhelmi et al., 2011; Ramireddy et al., 2018). Through transgenic approaches, the overexpression of IPT has been done in wheat, engendering a higher CK content with delayed leaf senescence and NR activity, which are positively associated with N remobilization and utilization process, respectively (Sýkorová et al., 2008; Masclaux-Daubresse et al., 2010). However, no significant increase in grain yield was observed (Sýkorová et al., 2008). Meanwhile, CXK has been overexpressed in barley, leading to the breakdown of CK (Holubová et al., 2018). It leads to a larger root system without a significant yield sacrifice (Holubová et al., 2018). Interestingly, at the same time, the thousand-grain weight was shown to be reduced while increases in tiller and grain numbers and the higher accumulation of nutrients were recorded (Holubová et al., 2018). Accordingly, grain productivity is under sophisticated control *via* CK as a signal between roots and above-ground plant, and more investigation into the molecular level would merit future research on barley NUE.

## 5 Conclusion

In conclusion, it must be acknowledged that there is limited amount of research has examined NUE in barley, especially considering the below-ground performance. First, several classes of candidate NUE genes have been identified in barley while only a few have been proved to be associated with root traits such as NAC domain protein gene. Moreover, most identified root-related QTLs are not consistent across experiments due to statistical limitations, and biological knowledge gaps including phenotyping platforms and the understanding of relevant phenotypes. Given the advances in plant genomic areas such as whole-genome sequencing technologies and the development of meta-analysis algorithm of GWAS, a large gap still exists in the association between plant phenotypes and N levels. At morphological and anatomical levels, future studies should not be limited to root biomass and length but pay more attention to RSA traits and RCS. Moreover, more research into nodal roots is also highly demanded. Regarding physiological aspects, different N forms are absorbed *via* different transporters. Most NUE research focuses on investigating the effects of NO_3_^−^ transporters. Nevertheless, a more effective uptake of NH_4_^+^ and urea can be potentially desirable for barley plants adapting to a wider range of environments and reducing N losses from the system. Lastly, hormonal regulation is a sophisticated mechanism to be examined for communicating roots with above-ground plant responses.
