# Supplementary material for: Mini review: Targeting below-ground plant performance to improve nitrogen use efficiency (NUE) in barley
Source: Front Genet. 2023 Mar 2;13:1060304. doi: 10.3389/fgene.2022.1060304 (PMC10017981; doi:10.3389/fgene.2022.1060304)
Supplement: Supplementary file 1 [file Table1.DOCX]

Supplementary Table S1. Candidate gene**s** related to NUE in barley

| **N use process** | **Function categories** | **Gene annotation** | **Candidate gene** | **Chromosome** | **Related root traits** | **Reference** |
| --- | --- | --- | --- | --- | --- | --- |
| N uptake | N transporters | Protein NRT1/PTR FAMILY 8.3 | HORVU4Hr1G064820^*^ | 4 | Relative root dry weight | Karunarathne et al., 2020a |
|  |  | Sodium-coupled neutral amino acid transporter 1 | HORVU6Hr1G072350 | 6 | Relative root dry weight | Karunarathne et al., 2020a |
|  |  | Nitrate transporter 2 (high affinity) | HvNRT2.1^**^ | 3 | N.A | Han et al., 2016 |
|  |  |  | HvNRT2.2 | 6 | N.A | Han et al., 2016 |
|  |  |  | HvNRT2.3 | 6 | N.A | Han et al., 2016 |
|  |  |  | HvNRT2.4 | 6 | N.A | Han et al., 2016 |
|  |  |  | HvNRT2.5 | 6 | N.A | Han et al., 2016 |
|  |  |  | HvNRT2.6 | 6 | N.A | Han et al., 2016 |
|  |  |  | HvNRT2.7 | 7 | N.A | Han et al., 2016 |
|  |  | NRT2 partner protein (NAR2) | HvNAR2.1 | 6 | N.A | Han et al., 2016 |
|  |  |  | HvNAR2.2 | 5 | N.A | Han et al., 2016 |
|  |  |  | HvNAR2.3 | 6 | N.A | Han et al., 2016 |
|  |  | Ammonium transporter | HvAMT1.1 | 6 | N.A | Han et al., 2016 |
|  |  |  | HvAMT1.2 | 2 | N.A | Han et al., 2016 |
|  |  | Lysine histidine transporter | HVLHT1 | 7 | N.A | Han et al., 2016 |
|  |  |  | HVLHT2 | 7 | N.A | Han et al., 2016 |
|  |  |  | HVLHT3 | 7 | N.A | Han et al., 2016 |
|  |  | Aquaporins (AQPs) that transport urea | HvPIP1;1 | 2 | N.A | Hove et al., 2015 |
|  |  |  | HvPIP1;2 | 5 | N.A | Hove et al., 2015 |
|  |  |  | HvPIP1;3 | 6 | N.A | Hove et al., 2015 |
|  |  |  | HvPIP1;4 | 6 | N.A | Hove et al., 2015 |
|  |  |  | HvPIP1;5 | 6 | N.A | Hove et al., 2015 |
|  |  |  | HvPIP2;1 | 6 | N.A | Hove et al., 2015 |
|  |  |  | HvPIP2;2 | 2 | N.A | Hove et al., 2015 |
|  |  |  | HvPIP2;2a | 2 | N.A | Hove et al., 2015 |
|  |  |  | HvPIP2;3 | 2 | N.A | Hove et al., 2015 |
|  |  |  | HvPIP2;4 | 2 | N.A | Hove et al., 2015 |
|  |  |  | HvPIP2;5 | 2 | N.A | Hove et al., 2015 |
|  |  |  | HvPIP2;6 | 5 | N.A | Hove et al., 2015 |
|  |  |  | HvPIP2;10 | 7 | N.A | Hove et al., 2015 |
|  |  |  | HvTIP1;1 | 4 | N.A | Hove et al., 2015 |
|  |  |  | HvTIP1;2 | 3 | N.A | Hove et al., 2015 |
|  |  |  | HvTIP2;1 | 6 | N.A | Hove et al., 2015 |
|  |  |  | HvTIP2;2 | 2 | N.A | Hove et al., 2015 |
|  |  |  | HvTIP2;3 | 7 | N.A | Hove et al., 2015 |
|  |  |  | HvTIP4;2 | 3 | N.A | Hove et al., 2015 |
|  |  |  | HvNIP2;1 | 6 | N.A | Hove et al., 2015 |
|  |  |  | HvNIP2;2 | 7 | N.A | Hove et al., 2015 |
|  |  |  | HvNIP2;3 | N.A | N.A | Hove et al., 2015 |
| N assimilation | Converting N forms | Nitrate reductase | HvNR1 | 6 | N.A | Han et al., 2016 |
|  |  |  | HvNR2 | 6 | N.A | Han et al., 2016 |
|  |  | Ferredoxin-nitrite reductase | HvNiR1 | 6 | N.A | Han et al., 2016 |
|  |  |  | HvNiR2 | 2 | N.A | Han et al., 2016 |
|  | Amino acid biosynthesis | Asparagine-tRNA ligase | HORVU1Hr1G024420 | 1 | Relative root dry weight | Karunarathne et al., 2020a |
|  |  | Asparagine synthetase | HORVU1Hr1G092110 | 1 | Relative root dry weight | Karunarathne et al., 2020a |
|  |  |  | HvASN1 | 4 | N.A | Han et al., 2016 |
|  |  |  | HvASN4 | 5 | N.A | Han et al., 2016 |
|  |  | Asparaginase | HvASNase1 | 2 | N.A | Han et al., 2016 |
|  |  |  | HvASNase2 | 2 | N.A | Han et al., 2016 |
|  |  | Asparate aminotransferase | HvASP1 | 6 | N.A | Han et al., 2016 |
|  |  |  | HvASP2 | 1 | N.A | Han et al., 2016 |
|  |  |  | HvASP3 | 7 | N.A | Han et al., 2016 |
|  |  |  | HvASP4 | 3 | N.A | Han et al., 2016 |
|  |  |  | HvASP5 | 6 | N.A | Han et al., 2016 |
|  |  |  | HvASP6 | 5 | N.A | Han et al., 2016 |
|  |  |  | HvASP7 | 3 | N.A | Han et al., 2016 |
|  |  | Asparagine synthase | HvAS | 5 | N.A | Han et al., 2016 |
|  |  | Putative, Glutathione S-transferase family protein | HORVU3Hr1G098810 | 3 | Relative root dry weight | Karunarathne et al., 2020a |
|  |  | Glutathione reductase | HORVU6Hr1G051300 | 5 | Relative root dry weight | Karunarathne et al., 2020a |
|  |  | Glutamic-pyruvate transaminase (alanine aminotransferase; GPT) | HvAlaAT1-1 | 1 | N.A | Han et al., 2016 |
|  |  |  | HvAlaAT2-1 | 2 | N.A | Han et al., 2016 |
|  |  |  | HvAlaAT2-2 | 2 | N.A | Han et al., 2016 |
|  |  |  | HvAlaAT5-1 | 5 | N.A | Han et al., 2016 |
|  |  |  | HvAlaAT5-2 | 5 | N.A | Han et al., 2016 |
|  |  | Glutamate glyoxylate aminotransferase (GGT) | HvGGT1 | 1 | N.A | Han et al., 2016 |
|  |  |  | HvGGT2 | 4 | N.A | Han et al., 2016 |
|  |  | Glutamate dehydrogenase NAD(P)H | HvGDH1 | 5 | N.A | Han et al., 2016 |
|  |  |  | HvGDH2 | 3 | N.A | Han et al., 2016 |
|  |  |  | HvGDH3 | 2 | N.A | Han et al., 2016 |
|  |  |  | HvGDH4 | 3 | N.A | Han et al., 2016 |
|  |  | Glutamine synthetase | HvGS1 | 6 | N.A | Han et al., 2016 |
|  |  |  | HvGS2 | 4 | N.A | Han et al., 2016 |
|  |  |  | HvGS3 | 2 | N.A | Han et al., 2016 |
|  |  |  | HvGS4 | 4 | N.A | Han et al., 2016 |
|  |  |  | HvGS5 | 4 | N.A | Han et al., 2016 |
|  |  | Glutamate synthase (NADPH/Ferredoxin) | HvGOGAT1 | 3 | N.A | Han et al., 2016 |
|  |  |  | HvGOGAT2 | 2 | N.A | Han et al., 2016 |
|  |  | Glycolate oxidase (GOX) | HvGOX1 | 2 | N.A | Han et al., 2016 |
|  |  |  | HvGOX2 | 2 | N.A | Han et al., 2016 |
|  |  |  | HvGOX3 | 5 | N.A | Han et al., 2016 |
|  |  |  | HvGOX4 | 2 | N.A | Han et al., 2016 |
|  |  |  | HvGOX5 | N.A | N.A | Han et al., 2016 |
| N remoblization | Delay leaf senescence | Stay green protein gene | HvSGR1 | 5 | N.A | Han et al., 2016 |
|  | Cytokinin regulation | Phosphate-isopentenyl transferase (IPT) | HvIPT1 | 1 | N.A | Han et al., 2016 |
|  |  |  | HvIPT2 | 2 | N.A | Han et al., 2016 |
|  |  |  | HvIPT3 | 3 | N.A | Han et al., 2016 |
|  |  |  | HvIPT4 | 3 | N.A | Han et al., 2016 |
|  |  |  | HvIPT5 | 1 | N.A | Han et al., 2016 |
|  |  | Cytokinin oxidase (CKX) | HvCKX1 | 3 | N.A | Han et al., 2016 |
|  |  |  | HvCKX2 | 6 | N.A | Han et al., 2016 |
|  |  |  | HvCKX3 | 3 | N.A | Han et al., 2016 |
|  |  |  | HvCKX4 | 3 | N.A | Han et al., 2016 |
|  |  |  | HvCKX5 | 1 | N.A | Han et al., 2016 |
|  |  |  | HvCKX6 | 3 | N.A | Han et al., 2016 |
|  |  |  | HvCKX7 | 2 | N.A | Han et al., 2016 |
|  |  |  | HvCKX8 | 3 | N.A | Han et al., 2016 |
| Participate in all metabolism processes | Regulating various biological activities as transcription factor, signalling genes etc. | Transcription factor bHLH140 | HORVU1Hr1G033980 | 1 | Relative root length | Karunarathne et al., 2020a |
|  |  |  | HvHLHm1 | 4 | N.A | Han et al., 2016 |
|  |  |  | HvHLHm2 | 4 | N.A | Han et al., 2016 |
|  |  |  | HvHLHm3 | 4 | N.A | Han et al., 2016 |
|  |  |  | HvHLHm4 | 4 | N.A | Han et al., 2016 |
|  |  | WRKY DNA-binding protein 23 | HORVU1Hr1G092130 | 1 | Relative root dry weight | Karunarathne et al., 2020a |
|  |  | Receptor kinase 1 | HORVU1Hr1G007930 | 1 | Relative root dry weight | Karunarathne et al., 2020a |
|  |  | Protein kinase superfamily protein | HORVU1Hr1G094990 | 1 | Relative root dry weight | Karunarathne et al., 2020a |
|  |  | Zinc finger A20 and AN1 domain-containing stress-associated protein 9 | HORVU2Hr1G036250 | 2 | Relative root dry weight | Karunarathne et al., 2020a |
|  |  | BnaA01g30480D protein | HORVU3Hr1G116150 | 3 | Relative root dry weight | Karunarathne et al., 2020a |
|  |  | Serine/threonine-protein kinase ATM | HORVU3Hr1G000420 | 3 | Relative root dry weight | Karunarathne et al., 2020a |
|  |  | FAR1 family | HORVU3Hr1G098810 | 3 | Relative root dry weight | Karunarathne et al., 2020a |
|  |  | NAC domain protein | HORVU3Hr1G095880 | 3 | Relative root dry weight | Karunarathne et al., 2020a |
|  |  |  | HvNAC1 | 4 | N.A | Han et al., 2016 |
|  |  |  | HvNAC2 | 7 | N.A | Han et al., 2016 |
|  |  |  | HvNAC3 | 5 | N.A | Han et al., 2016 |
|  |  |  | HvNAC4 | 5 | N.A | Han et al., 2016 |
|  |  |  | HvNAC5 | 7 | N.A | Han et al., 2016 |
|  |  |  | HvNAM1 | 6 | N.A | Han et al., 2016 |
|  |  |  | HvNAM2 | 2 | N.A | Han et al., 2016 |
|  |  | MADS-box transcription factor family protein | HORVU3Hr1G095090 | 3 | Relative root dry weight | Karunarathne et al., 2020a |
|  |  | Sccinate dehydrogenase subunit 4 | HORVU3Hr1G098920 | 3 | Relative root dry weight | Karunarathne et al., 2020a |
|  |  | Replication protein a 32 kda subunit | HORVU3Hr1G030580 | 3 | Relative root dry weight | Karunarathne et al., 2020a |
|  |  | Lucine-rich repeat receptor-like protein kinase family protein | HORVU3Hr1G098610 | 3 | Relative root dry weight | Karunarathne et al., 2020a |
|  |  |  | HORVU7Hr1G002010 | 7 | Relative root dry weight | Karunarathne et al., 2020a |
|  |  | Transcription factor GTE9 | HORVU3Hr1G015740 | 3 | N.A | Karunarathne et al., 2020a |
|  |  | Auxin-induced protein 5NG4 | HORVU4Hr1G073520 | 4 | Relative root dry weight | Karunarathne et al., 2020a |
|  |  | Disease resistance protein RPM1 | HORVU4Hr1G012820 | 4 | Relative root dry weight | Karunarathne et al., 2020a |
|  |  | Sucrose transporter 4 | HORVU5Hr1G000120 | 5 | Relative root dry weight | Karunarathne et al., 2020a |
|  |  | B protein | HORVU5Hr1G052590 | 5 | Relative root dry weight | Karunarathne et al., 2020a |
|  |  | Late embryogenesis abundant protein D-34 | HORVU5Hr1G005910 | 5 | Relative root dry weight | Karunarathne et al., 2020a |
|  |  | Serine/threonine-protein kinase | HORVU5Hr1G087040 | 5 | Relative root dry weight | Karunarathne et al., 2020a |
|  |  | Ethylene receptor | HORVU5Hr1G019650 | 5 | Relative root dry weight | Karunarathne et al., 2020a |
|  |  | Zinc finger CCCH domain-containing protein 19 | HORVU6Hr1G094650 | 6 | Relative root dry weight | Karunarathne et al., 2020a |
|  |  | Receptor-like protein kinase 1 | HORVU6Hr1G094650 | 6 | N.A | Karunarathne et al., 2020a |
|  |  | Basic helix-loop-helix (bHLH) DNA-binding superfamily protein | HORVU6Hr1G001200 | 6 | N.A | Karunarathne et al., 2020a |
|  |  | UDP-Glycosyltransferase superfamily protein | HORVU7Hr1G069690 | 7 | Relative root dry weight | Karunarathne et al., 2020a |
|  |  | 12-oxophytodienoate reductase 2 | HORVU7Hr1G036070 | 7 | Relative root dry weight | Karunarathne et al., 2020a |
|  |  | 2-oxoglutarate (2OG) and Fe(ii)-dependent oxygenase superfamily protein | HORVU7Hr1G122350 | 7 | Relative root dry weight | Karunarathne et al., 2020a |
|  |  | Thionin-like peptide | HORVU7Hr1G122800 | 7 | Relative root dry weight | Karunarathne et al., 2020a |
|  |  | F-box domain containing protein | HORVU7Hr1G114730 | 7 | Relative root dry weight | Karunarathne et al., 2020a |
|  |  | Ethylene-responsive transcription factor 11 | HORVU7Hr1G088790 | 7 | Relative root dry weight | Karunarathne et al., 2020a |
|  |  | Sucrose | HvPKABA1 | 2 | N.A | Han et al., 2016 |
|  |  | Heterotrimeric G-Protein | HvDEP1 | 5 | N.A | Han et al., 2016 |
|  |  |  | HvRGA1 | 7 | N.A | Han et al., 2016 |
|  |  |  | HvRGB1 | 4 | N.A | Han et al., 2016 |
|  |  | Mitogen-activate kinasekinase (MKK) | HvSMG1 | 6 | N.A | Han et al., 2016 |
|  |  |  | HvSMG2 | 5 | N.A | Han et al., 2016 |
|  |  | Non-fermenting-1 relatedkinases (SnRK) | HvPKABA2 | 2 | N.A | Han et al., 2016 |
|  |  |  | HvPKABA3 | 4 | N.A | Han et al., 2016 |
|  |  |  | HvPKABA4 | 5 | N.A | Han et al., 2016 |
|  |  |  | HvPKABA5 | 2 | N.A | Han et al., 2016 |
|  |  |  | HvPKABA6 | 5 | N.A | Han et al., 2016 |
|  |  |  | HvPKABA7 | 2 | N.A | Han et al., 2016 |
|  |  |  | HvPKABA8 | 3 | N.A | Han et al., 2016 |
|  |  |  | HvPKABA9 | 1 | N.A | Han et al., 2016 |
|  |  | Early nodulin like protein | HvEND93-1 | 7 | N.A | Han et al., 2016 |
|  |  |  | HvEND93-2 | 7 | N.A | Han et al., 2016 |
|  |  |  | HvEND93-3 | 6 | N.A | Han et al., 2016 |
|  |  | Cell wall invertase | HvCIN1 | 4 | N.A | Han et al., 2016 |
|  |  |  | HvCIN2 | 2 | N.A | Han et al., 2016 |
|  |  |  | HvCIN3 | 1 | N.A | Han et al., 2016 |
|  |  | DNA-binding One Zinc Finger (DOF) | HvDOF1 | 5 | N.A | Han et al., 2016 |
|  |  |  | HvDOF2 | 2 | N.A | Han et al., 2016 |
|  |  |  | HvDOF3 | 5 | N.A | Han et al., 2016 |
|  |  |  | HvDOF4 | 1 | N.A | Han et al., 2016 |
|  |  |  | HvDOF5 | 7 | N.A | Han et al., 2016 |
|  |  | Nuclear factor Y (NFY) | HvNF-YB2.1 | 1 | N.A | Han et al., 2016 |
|  |  |  | HvNF-YB2.2 | 3 | N.A | Han et al., 2016 |
|  |  |  | HvNF-YB2.3 | 2 | N.A | Han et al., 2016 |
|  |  | Aberrant panicle organization | HvAPO1 | N.A | N.A | Han et al., 2016 |
|  |  |  | HvFBX94 | 5 | N.A | Han et al., 2016 |
|  |  |  | HvFBX258 | 2 | N.A | Han et al., 2016 |
|  |  | Ferredoxin NADP(H) reductase | HvFNR1 | 7 | N.A | Han et al., 2016 |
|  |  |  | HvFNR2 | 5 | N.A | Han et al., 2016 |
|  |  |  | HvFNR3 | 6 | N.A | Han et al., 2016 |
| Unknown | Unknown | Unknown fuction | HORVU3Hr1G096720 | 3 | Relative root dry weight | Karunarathne et al., 2020a |
|  |  | Unknown fuction | HORVU4Hr1G012940 | 4 | Relative root dry weight | Karunarathne et al., 2020a |
|  |  | Undescribed protein | HORVU5Hr1G052600 | 5 | Relative root dry weight | Karunarathne et al., 2020a |
|  |  | Unknown fuction | HORVU6Hr1G051370 | 6 | Relative root length | Karunarathne et al., 2020a |
|  |  | Unknown fuction | HORVU6Hr1G094720 | 6 | Relative root dry weight | Karunarathne et al., 2020a |
|  |  | Undescribed protein | HORVU7Hr1G705780 | 7 | Relative root dry weight | Karunarathne et al., 2020a |

*Genes listed as gene IDs are the ones produced from GWAS study

**Genes listed as gene
